# Identification of a Highly Conserved Epitope on Avian Influenza Virus Non-Structural Protein 1 Using a Peptide Microarray

**DOI:** 10.1371/journal.pone.0149868

**Published:** 2016-03-03

**Authors:** Jiashan Sun, Xiurong Wang, Xuexia Wen, Hongmei Bao, Lin Shi, Qimeng Tao, Yongping Jiang, Xianying Zeng, Xiaolong Xu, Guobin Tian, Shimin Zheng, Hualan Chen

**Affiliations:** 1 College of Veterinary Medicine, Northeast Agricultural University, Harbin, China; 2 Animal Influenza Laboratory of the Ministry of Agriculture and State Key Laboratory of Veterinary Biotechnology, Harbin Veterinary Research Institute, Chinese Academy of Agricultural Sciences, Harbin, China; University of Alabama at Birmingham, UNITED STATES

## Abstract

Avian influenza virus (AIV) non-structural protein 1 (NS1) is a multifunctional protein. It is present at high levels in infected cells and can be used for AIV detection and diagnosis. In this study, we generated monoclonal antibody (MAb) D7 against AIV NS1 protein by immunization of BALB/c mice with purified recombinant NS1 protein expressed in *Escherichia coli*. Isotype determination revealed that the MAb was IgG_1_/κ-type subclass. To identify the epitope of the MAb D7, the NS1 protein was truncated into a total of 225 15-mer peptides with 14 amino acid overlaps, which were spotted for a peptide microarray. The results revealed that the MAb D7 recognized the consensus DAPF motif. Furthermore, the AIV NS1 protein with the DAPF motif deletion was transiently expressed in 293T cells and failed to react with MAb D7. Subsequently, the DAPF motif was synthesized with an elongated GSGS linker at both the C- and N-termini. The MAb D7 reacted with the synthesized peptide both in enzyme-linked immunosorbent assay (ELISA) and dot-blot assays. From these results, we concluded that DAPF motif is the epitope of MAb D7. To our knowledge, this is the first report of a 4-mer epitope on the NS1 protein of AIV that can be recognized by MAb using a peptide microarray, which is able to simplify epitope identification, and that could serve as the basis for immune responses against avian influenza.

## Introduction

Avian influenza (AI) is an infectious disease caused by the avian influenza virus (AIV), an RNA virus of the *Orthomyxoviridae* family [1]. AI is a highly contagious disease that can cause devastating effects in poultry. Various strategies ranging from vaccination to stamping out were utilized to control outbreaks [2]. Because of the low fidelity of the RNA-dependent RNA polymerase and the segmented nature of the viral genome, AIV has an exceptionally high probability of mutation during replication. Thus, many antigenic and genetic variants tend to emerge during virus circulation in the field. Based on the antigenic properties of the two surface glycoproteins, AIV can be classified into 16 subtypes of HA (i.e., H_1-16_) and 9 subtypes of NA (i.e., N_1–9_). AIV strains are further classified as low or highly pathogenic, based on certain genetic features or the severity of the illness they cause in intravenously inoculated young chickens in the laboratory. Occasionally, viruses are transmitted from wild aquatic birds to domestic poultry. Infections with these viruses can also occur in humans; however, the infections are highly species-specific. Since 1997, highly pathogenic viruses have crossed the species barrier to infect humans, causing serious disease in both man and poultry in Hong Kong, China. In late 2003, an outbreak of the highly pathogenic H5N1 AI occurred in south-east Asia [3]. AIV is considered one of the most threating viruses to the poultry industry around the world, making this virus a critical human health risk and of concern to health organizations.

AIV contains a single-stranded, negative sense, segmented RNA genome that consists of eight segments of viral RNA (vRNA) that encode 11 known proteins [4]. RNA segment eight of AIV encodes two different nonstructural proteins (i.e., NS1 and NS2), the mRNAs of which are transcribed off different reading frames. NS1 has a strain-specific length of 230–237 aa and an approximate molecular mass of 26 kDa [5]. It is a non-essential virulence factor and can be detected in the cytosol of the infected cells [6]. NS1 protein is multifunctional and significantly involved with the protein-RNA and protein-protein interactions [7]. It has two functional domains—dsRNA-binding domain (RBD) and effector domain (ED)[7]—which are essential for intracellular and extracellular interactions. This protein is unique and plays a role as either an inhibitor or an activator through the association of other internal activator factors or viral proteins in the virus life cycle [8].

Antigenic epitopes of influenza A virus have been studied for decades, not only as an immune response model system but also for their medical importance [9]. Numerous epitopes from various proteins of influenza virus have been identified in humans and mice [10]. Despite extensive research conducted on mapping epitopes of influenza in mammals, the understanding of epitopes from various proteins of influenza in chickens, and information regarding the NS1 protein is very limited [11]. Because NS1 is present in high levels in the infected cells, it serves as a good target for detection and diagnosis of productive AIV infection. Thus, we sought to generate broadly cross-reactive yet highly NS1-specific monoclonal antibodies (MAbs) that could be useful for characterizing this important viral protein from a wide range of viral subtypes.

Several experimental techniques are currently available for mapping the antigenic epitopes, including synthetic peptides-enzyme-linked immunosorbent assay (ELISA) assays and random phage display [12]. However, it is impractical to use these techniques on a genomic scale due to the high cost and immense effort involved. Recently, peptide microarrays that are useful for miniaturized high-throughput, high-content immunoassays have been developed [13–15]. The substitution of ELISA or phage display with a synthetic peptide microarray is a straightforward approach to map epitopes. In this study, a peptide microarray was used to identify epitopes recognized by a MAb against the NS1 protein of AIV MAb.

## Materials and Methods

### Ethics statement

Care of laboratory animals and animal experimentation were conducted according to animal ethics guidelines and approved protocols. All animal studies were approved by the Review Board of Harbin Veterinary Research Institute of the Chinese Academy of Agricultural Sciences and the Animal Care and Use Committee of Heilongjiang Province (SYXK (Hei) 2012–2067).

### Virus and cells

A/Duck/Guangdong/S1322/2010(H5N1) (DK/GD/S1322) was isolated from a healthy duck in the live poultry market during our routine surveillance. The swab sample was handled by virus isolation with embryonated SPF chicken eggs. This NS1 sequence is a 100% match with nonstructural protein 1 [Influenza A virus (A/grey heron/HK/861.1/2002(H5N1))] (GenBank: AAT73445.1). The myeloma cells SP2/0 and 293T cells were both cultured in Dulbecco’s modified Eagle’s medium (DMEM, GIBCO, Grand Island, NY, USA) in a humidified 5% CO_2_ atmosphere at 37°C supplemented with 10% (v/v) fetal bovine serum (GIBCO). Sf9 cells were maintained in Grace's Insect Medium (GIBCO) also with 10% (v/v) fetal bovine serum. All media were supplemented with 10,000 μg/mL of streptomycin and 10,000 units/mL of penicillin.

### Antigen preparation

The AIV NS1 protein was used as an antigen for the generation of MAbs, which was expressed in *Escherichia coli* BL21 (DE3) according to the following description. The AIV NS1 gene was amplified with reverse transcription polymerase chain reaction (RT-PCR) from the RNA of the DK/GD/S1322 virus and cloned into the expression vector pET-30a (Novagen, Darmstadt, Germany). All resulting recombinant plasmids were validated by restriction analysis and sequencing. The positive recombinant plasmid was named as pET-30a-NS1. The expression plasmid was transformed into BL21 (DE3) competent cells followed by the addition of 1 mM isopropyl-D-thioga-lactopyranoside (IPTG) for induction. The expressed NS1 proteins were purified using a Ni-NTA kit (Novagen), and the purified NS1 protein was stored at -80°C.

### Production of hybridoma cell secreting monoclonal antibodies

All mice were maintained in the animal facility at Harbin Veterinary Research Institute under standard conditions prescribed by the Institutional Guidelines. The study protocol was approved by the Institutional Animal Care and Use Committee. Six-week-old female BALB/c mice were subcutaneously immunized with 100 μg of the purified AIV NS1 antigen emulsified with an equal volume of complete Freund’s adjuvant (Sigma, St. Louis, MO, USA). Two boosters of incomplete Freund’s adjuvant (Sigma) emulsified antigen were administered at two-week intervals. Two weeks after the third immunization, the mice were intraperitoneally boosted with 100 μg of antigen alone. Three days later, immunized spleen cells were fused with SP2/0 myeloma cells using 50% (wt/vol) polyethylene glycol (Sigma), and hybridoma cells were cultured in DMEM plus HAT media supplement (Sigma). After four replacements of HT media supplement (Sigma), the hybridomas suspension was screened for positive clones using an indirect enzyme-linked immunosorbent assay (iELISA). A total of 0.4 μg/well of NS1 protein was coated on ELISA plates at 4°C overnight, and the plates were blocked by 5% skim milk at 37°C for 2 h. After washing three times in PBST, the hybridomas suspension was added to each well and incubated at 37°C for 1 h. A total of 100 μL/well of horseradish peroxidase (HRP)-conjugated goat anti-mouse IgG (Sigma) secondary antibodies at a dilution of 1:5000 in PBST was added and incubated at 37°C for 1 h. Then, HRP enzyme activity was determined and stopped by 2 M H_2_SO_4_, and the OD_490nm_ value was measured by BioRad 96-well plate reader (iMark, Bio-Rad Laboratories, Hercules, CA). The hybridoma-producing MAb against NS1 protein was subcloned three times by limiting dilution of the cells. Ascitic fluid was prepared by intraperitoneal injection of BALB/c mice with 10^6^ hybridoma cells. Antibody subtype identification was performed using an SBA Clonotyping System/HRP Kit (Southern Biotech Associated, Birmingham, AL, USA).

### SDS-PAGE and western blot

The purified NS1 fusion protein was subjected to 12% sodium dodecyl sulfate-polyacrylamide gel electrophoresis (12% SDS–PAGE). The gel was either stained with Coomassie blue staining solution or electrophoretically transferred to a nitrocellulose membrane (pore size: 0.2 μm, thickness: 4.0–7.5 mils, PALL, Port Washington, NY, USA). After blocking with 5% nonfat milk in PBS overnight at 4°C, the membrane was incubated with MAb D7 (diluted 1:2,000 in PBS) at 37°C for 1 h, washed three times with PBS containing 0.05% (w/v) Tween 20 (PBST, pH 7.4), and probed with a 1:5,000 dilution of HRP-conjugated goat anti-mouse IgG (Sigma, St. Louis, MO, USA) at 37°C for 1 h. Reactivity was visualized with the substrate 3, 3'-diaminobenzidine (DAB, Sigma).

### Indirect immunofluorescence assay

The NS1 gene was amplified using primer pairs NS1-pU (5′- ACACGAGCTCATGGATTCCAACACTGTG-3′) and NS1-pL (5′- CCGCTCGAGTCAAACTTCTGACTCAATTG-′3) from the pET-30a-NS1 and cloned into vector pCAGGS with chicken β-actin/rabbit β-globin hybrid promoter (AG) and the human CMV-IE enhancer in various mammalian cells. The positive clone was named pCAGGS-NS1 and was transfected into sf9 cells with Lipofectamine2000 (Invitrogen, Carlsbad, CA, USA), and the pCAGGS vector was used as a negative control. The transfected cells were fixed with 4% paraformaldehyde overnight at 4°C and then air dried. Next, 50 μL/well of anit-NS1 MAb was added at a 1:200 dilution in PBS for 1 h at 37°C. Sera collected from AIV immunized and unimmunized mice were used as positive and negative controls, respectively. After washing with PBS, 50 μL/well FITC-conjugated goat anti-mouse IgG (Sigma) at a 1:200 dilution were added and incubated for 1 h at 37°C. Plates were washed three times with PBS and observed under a Carl Zeiss Vision microscope (ZEISS Axio Observer D1, wavelength: 488 nm).

### Mapping epitopes by peptide microarray

The NS1 gene sequence deduced protein sequence is as follows: GSGSGSGSGMDSNTVSSFQVDCFLWHVRKRFADQELGDAPFLDRLRRDQKSLRGRGNTLGLDIETATRAGKQIVERILEEESDEALKMPASRYLTDMTLEEMSRDWFMLMPKQKVAGSLCIKMDQAIMDKTIILKANFSVIFDRLETLILLRAFTEEGAIVGEISPLPSLPGHTGEDVKNAIGVLIGGLEWNDNTVRVSETIQRFAWRSSDEDGRLPLPPNQKRKMARTIESEVGSGSGSGSG.

The C- and N-termini were elongated by a neutral (GS)_4_ flexible peptide to avoid truncated peptides, and this process had a slight impact on the linked sequences. The protein sequence was then translated into 15-mer peptides with a peptide-peptide overlap of 14 amino acids. The resulting peptide microarray covered 225 different antigen-derived peptides printed in double spots (450 peptides in total). Each array was framed by Flag and HA control peptides.

After 30 min of pre-swelling in standard buffer (PBS, pH 7.4 + 0.05% Tween 20) and 30 min in blocking buffer (Rockland blocking buffer MB-070), the peptide microarray was incubated with the secondary antibody (goat anti-mouse IgG (H+L) IRDye680) at a dilution of 1:5000 for 1 h at room temperature to analyze background interactions with the antigen-derived peptides.

Afterward, the peptide microarray was again washed 2×1 min with standard buffer, swelled for 30 min in standard buffer, and incubated overnight at 4°C with the mouse MAb ascites fluids at a dilution of 1:1000. Repeated washing in standard buffer (2×1 min) was followed by incubation for 30 min with the secondary antibody at a dilution of 1:5000 at room temperature. After 2×1 min washing in standard buffer, the microarray was rinsed with Millipore water, air dried and scanned.

### Antigen epitope analysis using peptide-coated plates

Peptide of amino acid sequence GSGSDAPFGSGS used in the ELISA was synthesized by the Harbin Boshi biological technology company according to the results of mapping epitopes with a peptide microarray. Wells were coated overnight at 4°C with 100 μL of peptides that were diluted to 2.75 pg/mL with 0.1 M carbonate-bicarbonate (CBS, pH 9.6). The solution was removed, and the wells were washed three times with PBST. The wells were blocked with 200 μL of 1% bovine serum albumin (BSA) for 2 h at 37°C and then washed with PBST three times. MAbs were added and incubated at 37°C for 1 h. After washing three times, 100 μL/well of HRP-conjugated goat anti-mouse IgG antibody at a dilution of 1:5000 in PBST was added and incubated at 37°C for 1 h. Finally, HRP enzyme activity was determined by o-phenylenediaminedihydrochloride (OPD) and the OD was measured at 490 nm using a BioRad 96-well plate reader (iMark).

### Dot-blot immunoassay

The peptide GSGSDAPFGSGS was synthesized with standard F-moc-solid phase peptide synthesis. According to an assessment with high-performance liquid chromatography (HPLC), the purity of the peptides was >95%. In dot-blot assays, a nitrocellulose membrane was pre-treated for 10 min with 2.5% glutaraldehyde. Peptides (25 μg, 10 μg, 4 μg, 1.6 μg, and 0.64 μg) were spotted on the activated membranes and left to dry at room temperature. After blocking nonspecific binding with 5% skimmed milk, mouse anti-NS1 MAb D7 was added and incubated at 37°C for 1 h. After washing three times, infrared-labeled goat anti-mouse IRDye 700 secondary antibodies (Li-CorBiosciences, Lincoln, NE, USA) were added to bind to the MAb D7. The bound complex was detected using the Odyssey Infrared Imaging System (Li-CorBiosciences).

### Reactivity of deletion epitope of NS1 protein in 293T cells with MAbs

From the pCAGGS-NS1 plasmid, a deleted 4-mer (DAPF) mutant named pCAGGS-NS1ΔDAPF was generated by PCR mutagenesis using primers. All plasmids were purified from bacteria by using an AXYGEN plasmid DNA preparation kit. To achieve transient expression of AIV NS1 protein, 293T cells in poly-D-lysine coated 6-well plates were transfected with either pCAGGS-NS1 plasmid or the truncated mutant pCAGGS-NS1ΔDAPF using Lipofectamine2000 according to the manufacturer's instructions. After 36 h of incubation at 37°C, the transfected cells in 6-well plates were fixed with 4% paraformaldehyde overnight at 4°C. The cells were incubated with anti-NS1 MAb ascitic fluid (dilution 1:100) for 30 min at 37°C and then washed with PBS before incubation with sheep anti-mouse FITC conjugates and DAPI. The cells were subsequently imaged by laser scanning confocal microscopy (TCS SP5, Leica Microsystems GmbH, Wetzlar, Germany).

### Homology analysis

To investigate the conservation of the identified linear epitope among AIV viruses, all sequences available in the NCBI Influenza Virus Sequence Database were aligned using Clustal X 1.83 software.

## Results

### Generation and characterization of MAb D7

**Fig 1:**
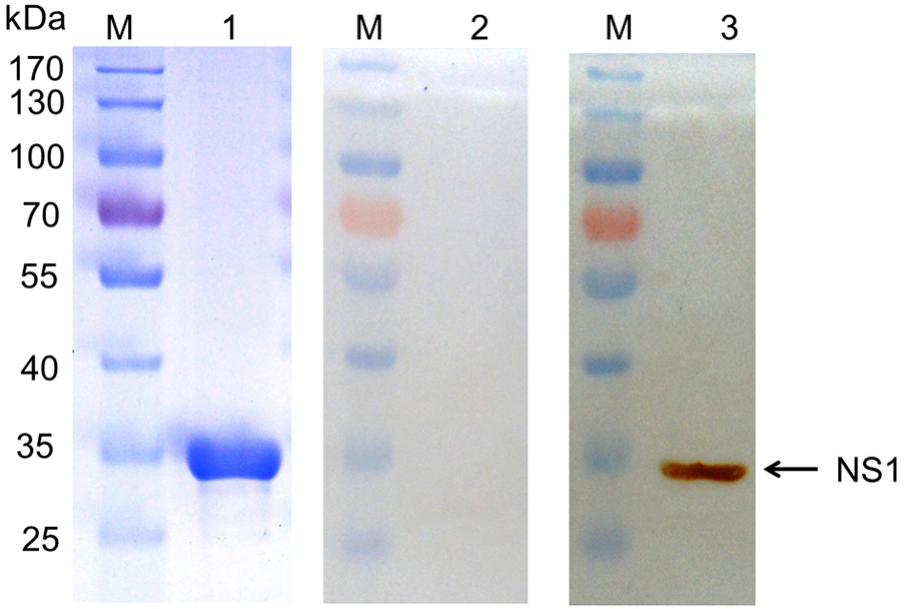
SDS-PAGE and Western blot analysis of purified recombinant NS1 protein expressed in *E*. *coli*. Lane M, molecular mass markers; lane 1, SDS-PAGE analysis of purified recombinant NS1 protein; Lane 2, the recombinant NS1 protein was analyzed by western blotting using SPF chicken serum for the negative control. Lane 3, western blotting of the purified recombinant NS1 protein, with chicken anti-AIV antibody and horseradish peroxidase (HRP)-labeled sheep anti-chicken IgG H+L as the first and second antibody, respectively. The arrowhead indicates the position of recombinant NS1 protein (approximate molecular mass of 30 kDa).

SDS-PAGE indicated that the purified recombinant NS1 protein had a single protein band of approximately 30 kDa, as expected. Western blot analysis revealed that this NS1 protein binds specifically to the serum collected from AIV-infected chickens (Fig 1). Thus, the NS1 protein was used as antigen for mice immunization. Three weeks after cell fusion, the hybridoma cell lines secreting anti-NS1 antibody were screened by means of an ELISA. One MAb D7 against AIV NS1 protein was generated. Isotype determination showed that the MAb D7 was a subclass IgG_1_/κ-type.

**Fig 2:**
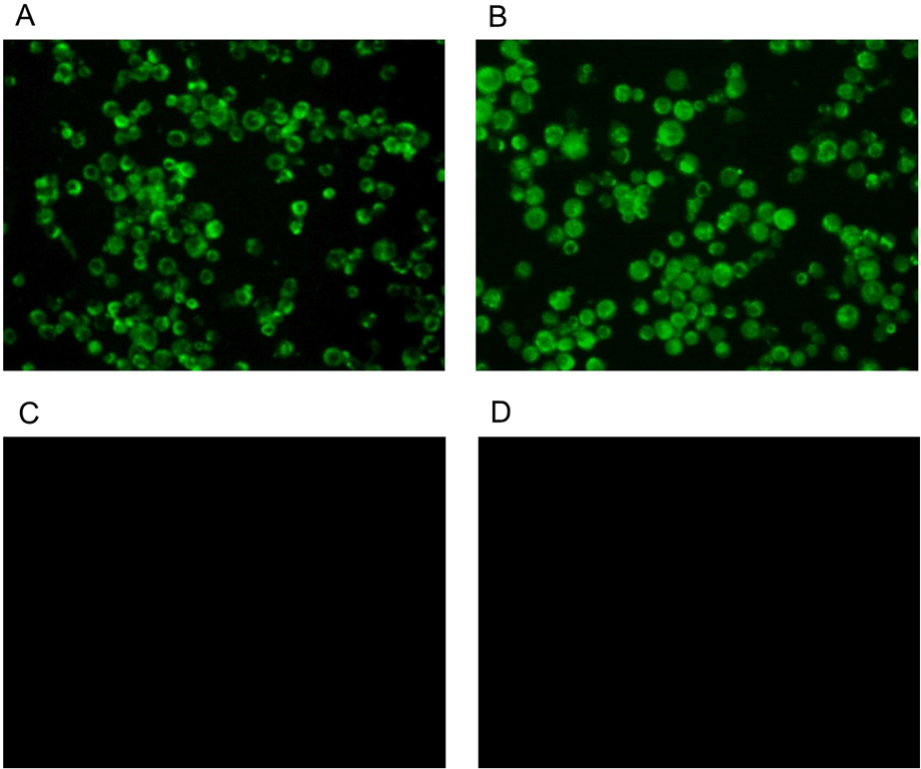
The MAb D7 is reactive to the eukaryotic expression recombinant NS1 protein. After 36 h of transfection, the sf9 cells were fixed with 4% paraformaldehyde overnight at 4°C and then probed with anit-NS1 MAb, positive-immunized mouse serum and negative normal mouse serum for 1 h at 37°C. Bound antibodies were visualized using FITC-conjugated antibodies against mouse IgG (1:200 dilutions) under a fluorescence microscope. A strong fluorescence signal was observed with positive-immunized mouse serum (A) and anit-NS1 MAb D7 (B). Control mouse serum (C) and the transfected blank pCAGGS plasmid sf9 cell (D) were also included as a negative control.

Indirect immunofluorescence assay showed that MAb D7 reacted with the pCAGGS-NS1 plasmid transfected sf9 cells (Fig 2). In addition, Western blotting demonstrated that MAb D7 recognized the recombinant NS1 protein, indicating a linear recognition of the epitope by MAb. These results indicated that the epitope recognized by MAb D7 is highly conserved.

### Mapping epitopes by peptide microarray

**Fig 3:**
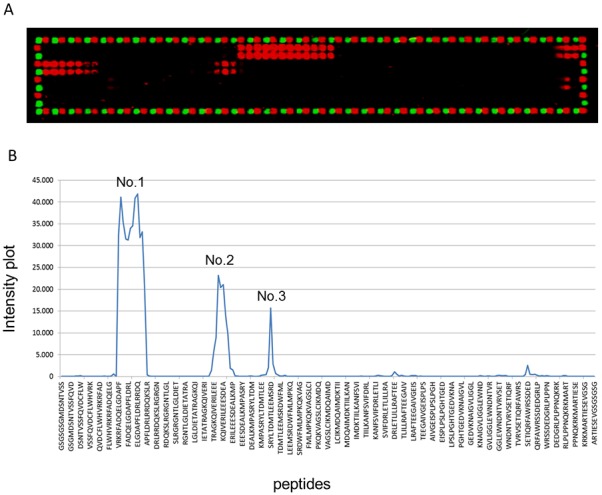
Mapping epitopes by peptide microarray. Incubation of the peptide microarray with the mouse monoclonal antibody at a dilution of 1:1000 was followed by staining with the secondary anti-mouse antibody and read-out at a scanning intensity of 4. Staining of FLAG and HA control peptides that frame the array gave rise to high and homogenous spot intensities at a green/red scanning intensity of 5/5 (A). The result of the fluorescence signal intensity and its representation of a deduced protein-sequence region (B). The intensity plot of all antigen-derived peptides on the microarray from the N- to the C-terminus clearly revealed three different epitopes (1–3) at high signal-to-noise ratios.

The peptide microarray was scanned with an Odyssey Imaging System (LI-COR Biosciences). Upon inspection, the MAb gave rise to clear substitution patterns, albeit with more than a single epitope, as expected for a monoclonal sample. Staining of Flag and HA control peptides that framed the arrays revealed high and homogeneous spot intensities (Fig 3A). Quantification of spot intensities and peptide annotation were performed with a PepSlide Analyzer. Based on the averaged medians of foreground intensities, an intensity map was generated as an intensity plot of the averaged spot intensities against the peptides on the microarray to visualize the signal-to-noise ratios and the overall spot intensities. The intensity plot revealed a clear interaction pattern with three different epitopes with high spot intensities and excellent signal-noise ratios (Fig 3B). The first epitope originated from peptides with the consensus motif DAPF (Fig 3B, No. 1). The second epitope was discovered with KQIVERILEEE as a consensus sequence with no similarity to the first motif (Fig 3B, No. 2). The third motif was based on the sequence RYLTDMTLEEMSR at weaker spot intensities. Given the gradually increasing and decreasing spot intensities of this peak, it could not be unambiguously determined whether the C- and N-terminal amino acids truly contributed to antibody binding (Fig 3B, No. 3). The number of real epitopes could have been reduced due to the similarity of RYLTDMTLEE to KQIVERILEE, of which the latter motif may also result from a cross-reaction of the antibody against the second motif IVERILEEE.

### Analysis of the binding of epitope 1 to MAb D7 against NS1

To confirm the reactivity of the MAb D7 to the DAPF epitope identified by the microarray, a DAPF peptide elongated with a neutral GSGS linker at both the C- and N-termini was synthesized as GSGSDAPFGSGS, which was used to determine its binding activity to MAb D7. ELISA results revealed that the peptide GSGSDAPFGSGS could be recognized by the MAb D7.

**Fig 4:**
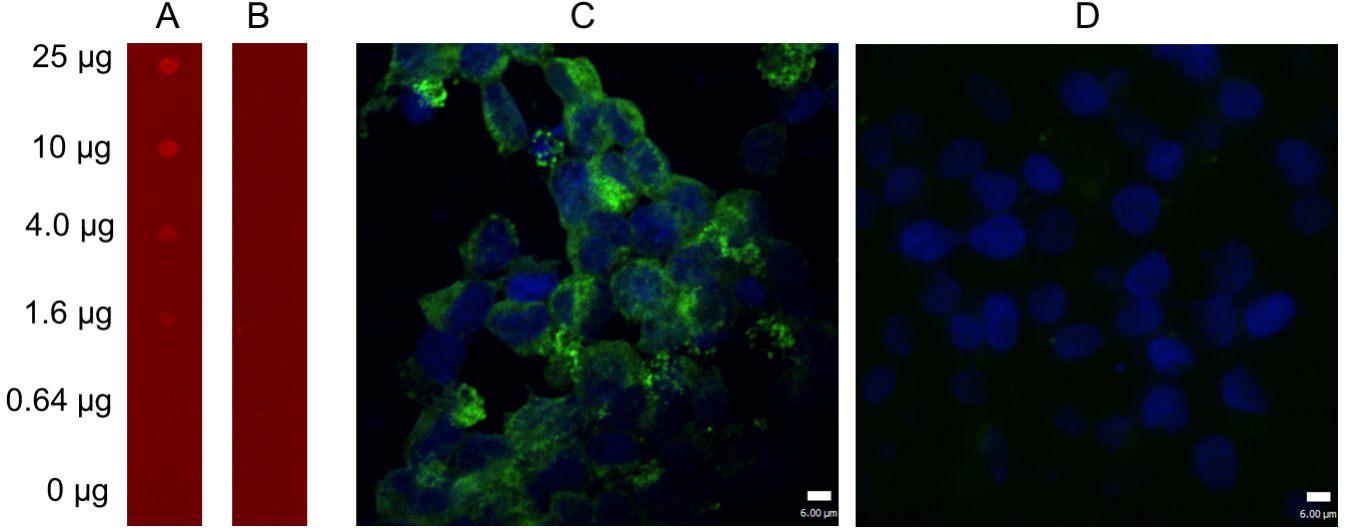
Analysis of binding of epitope 1 to MAb D7 against NS1. The results of A dot-blot immunoassay assay (A and B) and confocal laser scattering microscopy images (C and D). The peptide GSGSDAPFGSGS was spotted on the activated membranes and incubated with anti-NS1 MAb D7 (A) and control serum (B), staining of infrared-labeled goat anti-mouse IRDye 700 secondary antibody. The results showed that the peptide reacted to the MAb D7 in a concentration-independent manner. In addition, 293T cells were transfected with pCAGGS-NS1 (C) and pCAGGS-NS1-del-DAPF (D) using Lipofectamine 2000. After 36 h, 293T cells were fixed and probed with mouse anit-NS1 MAb ascitic fluid (dilution 1:100) followed by the FITC-conjugated goat anti-mouse antibody (green). Nuclei were counterstained with DAPI (blue). Scale bars are 6 μm.

In addition, the GSGSDAPFGSGS peptide reacted to the MAb D7 in a concentration-independent manner in dot-blot immunoassays (Fig 4A and 4B). Moreover, the NS1 protein without the DAPF epitope was expressed in 293T cells for further evaluation of the NS1 protein binding activity to the MAb D7. The 4 amino acids (DAPF) were deleted from the NS1 gene. This sequence cloned into a pCAGGS vector that was subsequently transfected into 293T cells to express the NS1 mutant protein. No green fluorescence was observed in 293T expressing recombinant NS1-mutant proteins lacking the DAPF epitope (Fig 4C and 4D), indicating that DAPF is the core epitope recognized by the MAbD7.

### Homology analysis

All sequences in the NCBI Influenza Virus Sequence Database were aligned using Clustal software to evaluate the conservation of the linear epitope recognized by MAb D7 among AIV viruses. Alignment indicated that the ^29^DAPF^32^ epitope was highly conserved among all AIV isolates available from GenBank; however, a few amino acid substitutions were identified.

## Discussion

NS1 has a strain-specific length of 230–237 amino acids. The NS1 protein is an extensively studied multifunctional protein that is commonly considered as a key viral component that allows inhibition against host immune responses [16]. Several studies have demonstrated that the viral gene encoding the NS1 protein is involved in different roles, including the regulation of interferon (IFN) expression [17], mRNA binding and the prevention of mRNA processing [18], and RNA silencing suppression [19, 20]. In this study, we generated a MAb D7 against AIV NS1 protein expressed in *E*. *coli*.

An epitope, also known as antigenic determinant, serves as the molecular basis of the immune response and immunological function [21, 22]. Such knowledge could lead to a better understanding of immunological mechanisms, particularly in the field of allergic response, by probing risk factors and advancing preventive strategies. The majority of identified influenza A virus epitopes are located on the HA protein and NP protein of the human influenza virus [23–25]. In the case of AIV, efforts have been made to map the epitopes of HA glycoprotein [26, 27] or the M protein [28], and epitopes of other proteins have rarely been reported. Therefore, the identification of epitopes for AIV-NS1 can provide important information for supplementing all the known whole virus epitope sites and further the understanding of immunological responses and pathogenesis in AIV infection.

Several methods have recently been developed for the identification of linear (or continuous) B-cell epitopes. The most common screening method is based on the use of a phage display random peptide library [29, 30], phage-ELISA, and software prediction methods. However, the existing methods for screening linear B-cell epitopes have limitations. In the present study, we developed an improved method using a peptide microarray to screen linear B-cell epitopes recognized by anti-NS1 proteins of AIV MAb. The results indicate that the MAb recognized the DAPF peptide sequence, which is located from AA29 to AA32. This strategy will help simplify epitope identification and could be used for virus-induced immune response evaluation.

All sequences in the NCBI Influenza Virus Sequence Database were aligned using Clustal software to investigate the conservation of the identified linear epitopes among AIV viruses. In total, 23,126 NS1 protein sequences are present in the database. A total of 99.8% of the sequences contained the DAPF epitope, among which 46 sequences contained amino acid substitutions. One sequence had amino acid deletions, whereas another sequence exhibited three amino acid substitutions (D to M, A to L, F to L). The remaining sequences had a unique amino acid substitution. Twenty-six sequences had an amino acid substitution at A: 8 sequences replaced A with T, 10 sequences replaced A with V, 7 sequences replaced A with S, and 1 sequence replaced A with D. Two sequences have a unique amino acid substitution at D: 1 sequence replaced D with V, and 1 sequence replaced D with A. Fifteen sequences had a unique amino acid substitution at F: 9 sequences replaced F with L, 3 sequences replaced F with S, 2 sequences replaced F with Y, and 1 sequence replaced F with V. One sequence replaced P with T (Table 1).

**Table 1:** Analysis the conservation of the identified epitope among AIV viruses using clustal software.

The position and quantity of amino acid substitutions (23,126)			
Single amino acid substitution (44)			
D-V (1)	-	-	-
D-A (1)	-	-	-
-	A-T (8)	-	-
-	A-V (10)	-	-
-	A-S (7)	-	-
-	A-D (1)	-	-
-	-	P-T (1)	-
-	-	-	F-L (9)
-	-	-	F-S (3)
-	-	-	F-Y (2)
-	-	-	F-V (1)
Three amino acids substitution (1)			
D-M	A-L	-	F-L
Deletion of one amino acid (1)			
Đ	-	-	-

The viruses of the sequences with these 44 amino acid substitutions were obtained from humans, avians and swine. Seventeen viruses were from humans, including the H1N1 subtype (12 strains) and H3N2 subtype (5 strains). Fourteen viruses were from swine, including the H1N1 subtypes (8 strains), H1N2 subtype (4 strains) and H3N2 subtype (2 stains). Fifteen other viruses were avian, and no representative strain was noted.

Many epitopes have been defined and mapped in the NS1 protein of influenza A majority subtypes. The following epitopes were previously mapped and included part of the epitope identified in this study: CFLWHVRKRVADQELGDAPF [31], ELGDAPFLDRLRRDQ, RVADQELGDAPFLDR [31], ADQELGDAPFLDRLRRD-QKS [32], DAPFLDRL, QELSDAPFL, DQDLGDAPFLDRLRRDQ, DQELSDAPF, RKQVADQDLGDAPFLDR [33, 34], GDAPFLDRLR and GDAPFLDRLRRDQKS-LRGRG [35]. These epitopes contained 8 to 20 amino acids, and all contained the DAPF motif. However, these results failed to indicate that the 4 amino acids (DAPF) were exclusively able to perform the epitope function. This result may be due to variations in testing methodology. To our knowledge, this is the smallest epitope, with only 4 amino acids, in the NS1 protein of AIV. The MAb and its epitope described in this study will facilitate further studies on the function of NS1 and diagnostic development for AIV detection. Our data could provide a basis for vaccine design and potential clinical applications. Our data may also be useful for further understanding of the antigenic structure of NS1 and its function in influenza virus pathogenesis.
